# Polar Bears from Space: Assessing Satellite Imagery as a Tool to Track Arctic Wildlife

**DOI:** 10.1371/journal.pone.0101513

**Published:** 2014-07-09

**Authors:** Seth Stapleton, Michelle LaRue, Nicolas Lecomte, Stephen Atkinson, David Garshelis, Claire Porter, Todd Atwood

**Affiliations:** 1 United States Geological Survey, Alaska Science Center, Anchorage, Alaska, United States of America; 2 Department of Fisheries, Wildlife and Conservation Biology, University of Minnesota, St. Paul, Minnesota, United States of America; 3 Department of Earth Sciences, University of Minnesota, Minneapolis, Minnesota, United States of America; 4 Department of Environment, Government of Nunavut, Igloolik, Nunavut, Canada; 5 Minnesota Department of Natural Resources, Grand Rapids, Minnesota, United States of America; Institut Pluridisciplinaire Hubert Curien, France

## Abstract

Development of efficient techniques for monitoring wildlife is a priority in the Arctic, where the impacts of climate change are acute and remoteness and logistical constraints hinder access. We evaluated high resolution satellite imagery as a tool to track the distribution and abundance of polar bears. We examined satellite images of a small island in Foxe Basin, Canada, occupied by a high density of bears during the summer ice-free season. Bears were distinguished from other light-colored spots by comparing images collected on different dates. A sample of ground-truthed points demonstrated that we accurately classified bears. Independent observers reviewed images and a population estimate was obtained using mark–recapture models. This estimate (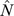: 94; 95% Confidence Interval: 92–105) was remarkably similar to an abundance estimate derived from a line transect aerial survey conducted a few days earlier (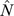: 102; 95% CI: 69–152). Our findings suggest that satellite imagery is a promising tool for monitoring polar bears on land, with implications for use with other Arctic wildlife. Large scale applications may require development of automated detection processes to expedite review and analysis. Future research should assess the utility of multi-spectral imagery and examine sites with different environmental characteristics.

## Introduction

The loss of Arctic sea ice has accelerated during recent years [1]–[3], with minimum sea ice extent reaching a record low during September, 2012. A nearly ice-free summer is now forecasted to occur as early as 2016 [4], . Such large-scale, precipitous environmental changes will be detrimental for many species dependent on sea ice habitats [6].

Despite potentially massive ecological impacts, regimes for monitoring wildlife remain deficient across large portions of the Arctic. For example, marine mammal assessment programs traditionally have used some combination of costly aircraft- or ship-based surveys and/or mark-recapture programs [7], [8], but the precision of resulting demographic estimates is often inadequate to detect trends in abundance [9]. Moreover, some areas are simply too inaccessible for routine monitoring. As such, baseline or long-term data are lacking for numerous species, precluding status and trend assessment and hindering management efforts. Walrus (*Odobenus rosmarus*) [10] and ribbon seals (*Histriophoca fasciata*) [11] are among the Arctic marine mammals currently classified as data deficient by the International Union for Conservation of Nature (IUCN). Likewise, data are insufficient to assess polar bear (*Ursus maritimus*) status across large portions of their range [12]; even in surveyed areas, monitoring intervals are often inadequate [13]. More frequent, systematic and efficient population surveys are needed to match the data needs of resource managers faced with a rapidly changing environment.

Recent advancements in satellite technology (resolutions of 0.5–5 m) have provided new tools for monitoring wildlife. Previous studies used satellite imagery to estimate abundance at Weddell seal (*Leptonychotes weddellii*) haul-outs [14] and emperor penguin (*Aptenodytes forsteri*) colonies in Antarctica [15]. Similarly, Platonov et al. [16] reported that polar bears, walrus and other marine mammals are visible on satellite imagery, but their findings are limited by an absence of ground-truthed data. Remote sensing affords access to vast expanses of otherwise inaccessible sites, at potentially reduced costs, without concerns about human safety and disturbance to wildlife.

Here, our goal was to evaluate the utility of high resolution satellite imagery to monitor Arctic wildlife, using polar bears as a case study. Whereas polar bears rank among the most studied large mammals globally, with capture datasets in some regions extending >30 years [17], most of that research has focused on a few easily accessible subpopulations. Polar bears are categorized as Vulnerable by the IUCN, largely owing to projected sea ice losses [18], but there is a paucity of population-level data across several broad regions. Additionally, the changing sea ice dynamics have led to shifts in the onshore distribution and abundance of polar bears [19]. Mitigating corresponding increases in human – bear conflicts requires an understanding of and ability to predict these distributional shifts. These issues highlight the need for efficient methods of population assessment that overcome logistical challenges, facilitate regular monitoring, and are consistent with the values of northern communities concerned about disturbance to wildlife.

## Methods

### Ethics Statement

This research was conducted under Wildlife Research Permit Number 2012-052 (Government of Nunavut). Aerial survey field protocols were approved by the Institutional Animal Care and Use Committee at the University of Minnesota (Permit Number 1207A17284).

### Study Area

We conducted our research in Foxe Basin, Nunavut, located in a seasonal ice region of the eastern Canadian Arctic. Recent comprehensive aerial surveys documented high densities of polar bears on relatively small islands (totaling <3,000 km^2^) in northern Foxe Basin with low topographic relief and no snow cover during the late summer, ice-free season [20]. As the ice melts across Foxe Basin, bears become stranded on small ice floes and eventually retreat to nearby land masses where they wait for ice to return. Hence, high densities of bears tend to accrue on land adjacent to late-melting ice, especially islands where dispersion is limited. We selected Rowley Island as our study site: its high density of bears during the ice-free season, contrasting dark landscape, and flat terrain provided an ideal setting to evaluate the utility of satellite imagery.

### Remote Sensing

We procured target satellite images of Rowley Island (∼1,100 km^2^) from DigitalGlobe, Inc. (WorldView-2 satellite, ∼0.5 m resolution at nadir; Quickbird, 0.65 m resolution), during early September, 2012. We compared these images to reference images to discriminate non-target objects from bears (∼2-m white objects visible on the target image but not the reference image). Reference imagery was acquired during August, 2009 and 2010 (WorldView-1; 0.5 m resolution) and August, 2012 (Quickbird; all satellite images are available for purchase through DigitalGlobe, Inc., http://www.digitalglobe.com.) We corrected all images for terrain (i.e., orthorectification). To account for any differences in sensor exposure settings and sun irradiance based on time of year and day, we calculated top-of-atmosphere reflectance (following [21]) using relevant metadata from the imagery (per band), earth-sun distance at time of acquisition, and sun elevation angle. We applied an additional histogram stretch to brighten darker, non-ice areas (identical for all images) in order to facilitate image comparison by human analysts. We used a Python script that leverages the open-source Geospatial Data Abstraction Library package for image manipulation and ArcGIS 10.1 (Environmental Systems Research Institute; Redlands, California, USA) to overlay target images on reference imagery.

Two independent observers visually identified potential polar bears on the September, 2012 image and recorded latitude and longitude. Observers initially reviewed imagery at a fixed scale of 1∶2,000 to 1∶3,000 and subsequently examined potential polar bears at multiple scales (up to ∼1∶250), and in comparison to reference images to help distinguish likely bears.

Following this independent review, the two observers jointly examined imagery to resolve uncertainties in identification of potential bears. We did not categorize an object as a “presumed bear” unless observers were in agreement and confident in that classification. We thus deleted some points from each observer's initial list of candidate polar bears, but observers did not add points to their respective sightings during this process. We treated each observer's review as an independent sampling period, enabling us to generate capture histories for mark-recapture analysis. We employed a full likelihood-based, closed population model [22], facilitating direct estimation of abundance and detection. We allowed detection probabilities to vary between observers and conducted modeling in Program MARK [23].

### Field Sampling

We used a helicopter (Bell 206L) survey to assess how well we distinguished polar bears from objects of similar size and color on imagery. We categorized 26 points on imagery as either polar bears or non-target, light-colored control points (e.g., rocks, foam on water surface), and we flew to these sites to confirm identity. We assumed that a bear had been present when the site had been photographed if 1) there was no rock or other feature that could be confused with a bear and 2) the site was not prone to ephemeral landscape features (e.g., not downwind of a pond that could have had white foam when the image was collected).

We also conducted a helicopter-based aerial survey to obtain a second population estimate of bears on Rowley Island. We could not directly compare polar bear sightings during this aerial survey (August 30 – September 1, 2012) with points on the target image (September 3) because bears moved in the 2–4 days that elapsed between events. However, we assumed that Rowley Island was a closed population during this short time frame, enabling us to compare abundance estimates derived by the two techniques.

**Figure 1:**
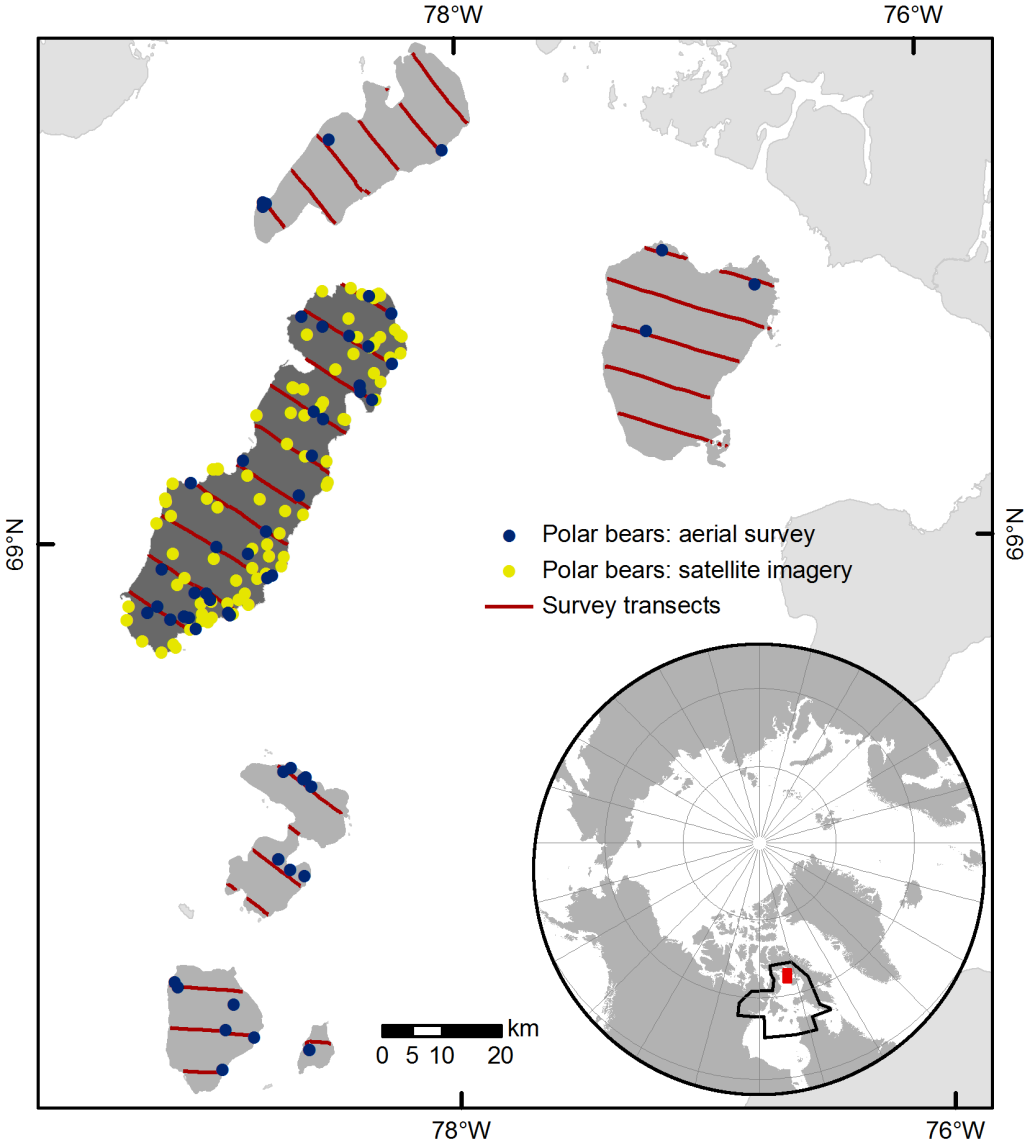
Polar bears detected with high resolution satellite imagery and during the helicopter-based aerial survey. Target imagery was acquired from Rowley Island (dark shade) in northern Foxe Basin, Nunavut with the WorldView-2 and Quickbird satellites on September 3, 2012. Transects were spaced at 7 km intervals during the aerial survey. The Foxe Basin polar bear subpopulation is outlined in black and the study area is shaded red in the inset.

We implemented mark-recapture distance sampling (MRDS) [24] protocols for abundance estimation. MRDS combines distance sampling with a double-observer platform; the double-observer data are incorporated in a mark-recapture modeling framework to explicitly test distance sampling's assumption of perfect detection at distance 0 [25] and inflate density estimates, if necessary. Here, bears observed by the pilot and front seat observer were considered marked, while those observed by rear seat observers were considered recaptured. We surveyed Rowley and nearby islands to obtain a sufficient sample for estimating the detection function. We oriented sampling transects perpendicular to each island's primary axis and extended them across island widths (Fig. 1). Transects were spaced at 7-km intervals, and we sampled at an above ground level altitude of 120 m (400 ft) and target airspeed of 160 km/h (85 knots). Flight parameters were based on previous overland aerial surveys of polar bears in the region [20]. We recorded flight paths and locations of polar bear sightings with a GPS, and we measured distances from transects to observations in a GIS (modified from [26]). We documented group size and recorded conditions that may have impacted detection (weather, lighting).

We conducted preliminary double-observer analyses with the Huggins model [27], [28], which suggested that detection on and near the transect line was nearly perfect. Hence, we analyzed data in the conventional and multiple covariate distance sampling engines of Program DISTANCE 6.0 [29]. We pooled sightings data from all islands to estimate a common detection function and used encounter rates and group sizes from Rowley Island to obtain an island-specific abundance estimate. We considered models with standard key functions and series expansion terms as well as covariate-based models. Because we could not reliably differentiate family groups on satellite imagery, our aerial survey estimate included only independent bears (i.e., excluded cubs or yearlings with their mother). We used Akaike's Information Criteria, adjusted for small sample sizes (AIC*_c_*) [30], for model selection.

## Results

### Remote Sensing

**Figure 2:**
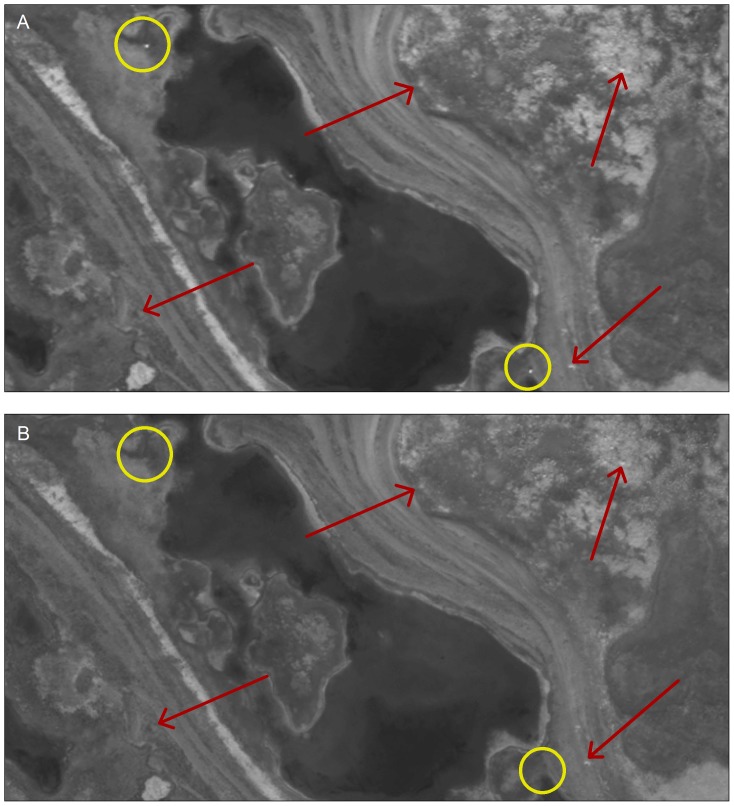
Example of high resolution satellite imagery used to detect polar bears. Imagery was procured from Rowley Island in Foxe Basin, Nunavut during late summer, 2012. The target imagery (a) was searched for polar bears, and the reference imagery (b) was used for comparison. Polar bears are present in the example target image but absent in the reference image (yellow circles). Landscape features that remain consistent between images, including rocks and substrate, are denoted with red arrows. Satellite imagery printed under a CC BY license, with permission from DigitalGlobe ©2013.

**Figure 3:**
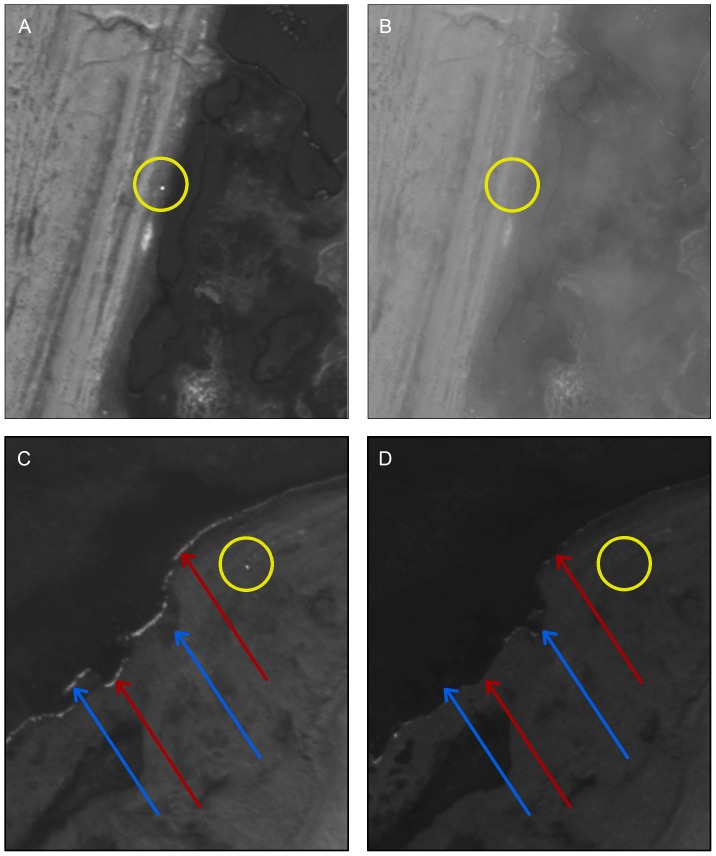
Clouds (top) and water conditions (bottom) are factors that may hamper detection of bears. Bear locations are indicated in target [(a) and (c)] and reference [(b) and (d)] images with yellow circles. Foam accumulating along the edges of water bodies and changes in water levels between target and reference images are indicated in the bottom pair of shots by red and blue arrows, respectively. Satellite imagery printed under a CC BY license, with permission from DigitalGlobe ©2013.

We detected 92 presumed bears on satellite images of Rowley Island (Figure 1) and documented likely family groups (adult females with cubs) on five occasions. The most highly supported model included separate detection probabilities for the two observers and yielded an abundance estimate of 94 (95% confidence interval: 92–105) independent bears. Individual detection probabilities varied greatly between the two observers (96% [95% CI: 83%–99%] and 42% [95% CI: 32%–52%]). Although it was generally straightforward to distinguish bears from other objects (Figure 2), landscape features and environmental characteristics sometimes complicated detection (Figure 3). About 12% of the reference imagery was obscured by clouds, and strong winds on the date of imagery collection created large expanses of foam along the banks of ponds that initially appeared to be bears, since they were absent from reference imagery. Additionally, some rocks reflected light differently between successive photos, requiring careful scrutiny to differentiate them from bears. However, joint review of imagery enabled us to correctly categorize all points that we ground-truthed via helicopter as presumed bears (n = 13) or inanimate objects (n = 13).

### Aerial Survey Abundance Estimation

During the helicopter aerial survey, we sighted 56 polar bear groups totaling 77 individuals along ca. 400 km of transects across all study islands; this included 33 groups (34 independent bears) during ca. 160 km of sampling on Rowley Island (Fig. 1). Despite a small number of detections, our data facilitated estimation of a robust detection function, and abundance estimates were consistent among the most highly supported models (Table 1). Our model-averaged [31] estimate of abundance (including models ΔAIC*_c_*<3) yielded 102 independent bears on Rowley Island (95% CI: 69–152).

**Table 1:** Summary of distance sampling analyses.

		Estimate (SE)			Goodness of Fit		
Model	ΔAIC*_c_*	ESW	p	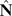	C-S	K-S	C-vM
Uniform/Cosine	0.00	1234 (65)	0.53 (0.028)	97 (17.8)	0.75	0.79	0.6
Half-normal/None	0.15	1151 (114)	0.50 (0.049)	104 (21.0)	0.75	0.94	0.8
Half-normal/VIS	1.12	1136 (114)	0.49 (0.049)	105 (21.3)	0.66	0.87	0.7
Half-normal/LIGHT	1.38	1112 (115)	0.48 (0.050)	108 (22.1)	0.55	0.91	0.8
Hazard/None	2.62	1201 (168)	0.52 (0.073)	100 (22.5)	0.53	0.89	0.8

Results of distance sampling analyses of a polar bear aerial survey conducted in northern Foxe Basin, Nunavut, Canada during August – September, 2012. Highly supported models (ΔAIC*_c_*<3) are presented. In the column Model, the key function is followed by adjustment terms or covariates (VIS =  visibility; poor/fair (e.g., glare, light fog or rain) or excellent; LIGHT =  light conditions; overcast, mostly cloudy, or partly cloudy/clear). ESW =  Effective strip width (meters). p =  Detection probability. 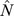 =  Abundance estimate. Goodness of Fit metrics: C-S =  Chi-squared; K-S =  Kolmogorov-Smirnov; C-vM =  Cramér-von Mises.

## Discussion

Satellite imagery shows promise as a means to quickly and safely monitor the abundance and distribution of polar bears using onshore habitats. We were able to discriminate among presumed bears and non-targets by comparing high resolution images collected at different points in time. The remarkable consistency between our estimates of abundance derived from imagery and established aerial survey techniques suggests that bear identification using imagery was quite accurate. We believe that the methods employed here (use of reference images, review by multiple observers to build consensus and generate capture histories, and estimation of abundance and detection probabilities via population models) provide a framework for other small-scale studies. However, applications at broader geographic scales may necessitate the development of automated image classification processes to expedite review and analysis. Our initial, independent review of imagery was tedious and required a combined 100 hours; this made it unrealistic to re-examine the images a second time (after our joint examination of points), and also made it difficult to recruit more observers. A reliable, automated process would greatly enhance the applicability of this technique.

Observers differed substantially in their abilities to detect bears with imagery. This finding was an unexpected but important result of this study; this did not diminish the robustness of our results, although precision would improve with higher detection for both observers. In our study, the two observers had vastly different levels of experience: one had several seasons of experience studying polar bears in this landscape during the ice-free season, whereas the other had extensive experience interpreting remote sensing imagery but no direct experience with polar bears. The observer with field experience had better detection of bears on the images, suggesting that familiarity with the study landscape and first-hand knowledge of bear biology and behavior (e.g., variation in color and body outline based on posture) greatly improved detection. Moreover, the observers searched imagery somewhat differently. We found that detection was higher when one regularly compared the target and reference images (one's eye was attracted to white spots on the target image not present on the reference image), rather than using the reference image to simply verify the presence of bears. These experiences suggest that explicit search protocols and a rigorous training program including individuals with relevant, on-the-ground experience with the target species will improve implementation of the technique and ensure appropriate search images.

The two abundance estimation techniques provided significantly different estimates of precision (coefficients of variation for line transect aerial survey: 20.4% versus satellite imagery: 2.5%). Distance sampling incorporates multiple variance components, including detection and encounter rates on sampled transects. Conversely, the satellite imagery modeling only includes a variance component for detection, since we reviewed imagery from the entire island. The very high detection probability of one imagery observer also contributed to this difference. Variance estimated from manual review of imagery would increase in applications in which observers have lower detection probabilities or if images provide less complete coverage of the study site.

Synchronizing collection of satellite imagery with visual surveys is not currently possible, since there is no assurance as to when the satellite's orbit will pass above the study area and if weather will be conducive to shooting imagery or conducting an aerial survey. This reality prohibits directly matching bears identified on photos with bears observed during an aerial survey. As such, absolute confirmation of presumed bears is impossible, and thus some false positives (i.e., inanimate objects classified as bears) or negatives are likely to occur.

Because one observer of the images had a very high detection probability (96%), we deemed it unnecessary to model potential sources of heterogeneity. However, future studies may be compelled to quantify variables potentially impacting detection. We hypothesize that environmental conditions including wind, light, and the presence of clouds and small onshore ice floes may affect detection (Fig. 3). Other prospective covariates may include bear reflectance values, bear size (i.e., pixels), reflectance values and complexity metrics for the surrounding landscape at multiple spatial scales, image exposure, and off-nadir angle at image collection [32].

We presumed that cubs were not consistently identifiable on imagery, given the resolution constraints. Their presence was suggested by multiple white spots of notably different sizes in a cluster (ca.<20 m). We detected only five likely family groups with imagery, whereas the nine family groups sighted on Rowley Island during aerial survey sampling suggest that there were ∼28 family groups present island-wide. The inability to reliably discern family groups poses some limits on the utility of imagery for demographic studies. However, the advent of higher resolution imagery (e.g., WorldView-3 platform, set to launch in 2014, will shoot at 0.3 m resolution at nadir) may permit differentiation of cubs, as well as improve detection of smaller species, in the future.

With minimal topographic relief and high densities of polar bears during late summer, our study island provided a model setting to test satellite imagery as a monitoring tool. Conditions elsewhere in the Arctic, however, are less ideal, and further technique development will be required to more broadly apply the technology. Priority research and development areas for polar bears should include assessing onshore sites with lower densities and more variable landscapes (e.g., higher topographic relief) and evaluating sampling intensities necessary to obtain reliable density estimates and distributional information. Additionally, multi-spectral imagery may better capture unique spectral signatures of the target species, thereby improving manual and automated detection in more challenging onshore environments. Multi-spectral imagery also may facilitate the detection of polar bears on sea ice, given the apparent spectral differences between bears and snow at short wavelengths (G. LeBlanc, National Research Council Canada and C. Francis, Environment Canada, unpublished data).

The success of this technique with polar bears suggests that satellite imagery would likely provide a useful means to inventory other megafauna as well. In the Arctic, darker species such as musk oxen (*Ovibos moschatus*) and caribou (*Rangifer tarandus*) may be readily detected against a snow-covered, springtime landscape. Whereas satellite imagery does not yield the same detail of information as traditional capture programs and aerial surveys, it has tremendous potential to provide coarse abundance and distribution data from sites otherwise too logistically challenging or costly to routinely access. The technology can open vast, remote regions to regular monitoring, facilitating the collection of data across species' ranges and at global scales. Understanding and predicting shifts in abundance and distribution of wildlife is critical to evaluating the ecological impacts of a rapidly changing climate. With archives dating back nearly a decade, imagery provides the opportunity to establish short-term longitudinal data.
